# Mediating Role of Emotional Intelligence in the Relationship between Anxiety Sensitivity and Academic Burnout in Adolescents

**DOI:** 10.3390/ijerph20010572

**Published:** 2022-12-29

**Authors:** José Jesús Gázquez Linares, María del Mar Molero Jurado, María del Carmen Pérez-Fuentes, África Martos Martínez, María del Mar Simón Márquez

**Affiliations:** 1 Department of Psychology, Universidad Autónoma de Chile, Providencia 7500912, Chile; 2 Department of Psychology, University of Almería, 04120 Almería, Spain; 3 Department of Psychology, Universidad Politécnica y Artística del Paraguay, Asunción 1628, Paraguay

**Keywords:** academic burnout, anxiety sensitivity, emotional intelligence, adolescence

## Abstract

(1) Background: Increased stress and study overload during adolescence could be related to academic burnout. Anxiety sensitivity of students seems to affect burnout levels, while emotional intelligence enables emotion management under stressful conditions. The objective of this study is to analyze the roles of anxiety sensitivity and emotional intelligence in academic burnout. (2) Methods: In this quantitative cross-sectional study conducted in Spain, the sample comprised 1287 high school students aged 14 to 18 who filled out the Maslach Burnout Inventory-General Survey, the Spanish version adapted for high school students of the Anxiety Sensitivity Index-3, and Brief Emotional Intelligence Inventory. (3) Results: Anxiety sensitivity was significantly higher in cynicism and emotional exhaustion. Furthermore, the mediation analysis showed that high anxiety sensitivity directly affected student exhaustion, cynicism, and efficacy levels. These effects were mediated mainly through stress management but also through the effect of stress management on mood, both emotional intelligence factors. (4) Conclusions: The academic changes that occur during high school hinder engagement and performance. Promoting the development of emotional skills would enable young people to manage their emotions when they become overwhelming and diminish their lack of interest and exhaustion in the classroom.

## 1. Introduction

### 1.1. Burnout in the Academic Context

One of the main social goals of education for adolescents is to attain a certain degree of well-being and adaptation in high school [1,2,3,4]. Going from elementary to high school usually generates a decrease in emotional engagement with school, and the new academic context is perceived as not pleasant or valuable. At the same time, anxiety about studies and overwork increases [5]. Similarly, abrupt changes associated with this stage, such as the heavy load of homework and the need to clarify their interests to choose their future path in education, can cause stress and even the development of burnout [6,7,8]. Exposure to minor stressors in adolescence is linked to various emotional and behavioral problems [9]. Academic burnout refers to the student’s lack of enjoyment or perceived value of learning, along with feelings of distress and being overwhelmed [5]. This construct consists of three factors, school exhaustion, cynicism about the value of school, and feeling unable to be successful at school [10]. Its incidence is very high among high school students [11].

Young people who show academic burnout feel unable to do any more than they already are, which generates a negative critical attitude, loss of interest, and academic motivation, along with doubt in their ability to complete their studies [12,13]. Noh et al. [14] found that high school students develop this syndrome when they feel emotionally drained by school demands and ineffective in doing their homework, which later generates an attitude of detachment and cynicism toward study and education. Thus, emotional exhaustion and low academic efficacy are the first signs of burnout, which, if not cut off, end up generating the last factor of academic exhaustion, which is cynicism.

Academic burnout is negatively related to student academic self-efficacy, motivation to learn, and academic engagement [15,16]. This syndrome has also been associated with the frequency of alcohol and cannabis use in young people who are just starting with it, and it has been postulated that it is a means of coping with exhaustion and school-related stress [17]. Other types of problematic behavior related to the development of academic burnout are excessive use of mobile phones, addiction to the Internet, and use of social networks [18,19,20]. School exhaustion is related to lower participation, educational goals, and classroom performance [21,22]. Its development has been postulated as a foregoer of lower academic performance [23].

### 1.2. Burnout and Anxiety Sensitivity

Stressful academic events increase the probability of high school students showing anxiety symptoms [24,25], especially the more vulnerable adolescents [26]. These symptoms, such as rapid heartbeat, sweating, or trouble concentrating, can be interpreted as an especially aversive feeling, which provokes avoidance of situations that generate them [27,28,29].

Anxiety sensitivity is the personal tendency to perceive sensations and symptoms of anxiety as harmful [30]. This variable has three dimensions related to beliefs that bodily sensations are potentially fatal (physical anxiety sensitivity), feeling that problems concentrating and restlessness could be dangerous (cognitive anxiety sensitivity), and beliefs that exteriorized anxiety symptoms will cause rejection or ridicule by others (social anxiety sensitivity) [31]. More vulnerability to stress and anxiety sensitivity has been shown to favor the appearance of exhaustion at school age [32], and students with high anxiety show more burnout symptoms [33].

According to previous research, students with high anxiety sensitivity levels have a cognitive bias in processing social and psychological stimuli [34]. This could be related to a strong feeling of threat and stress from school, which would end up generating burnout, as both factors (anxiety and depression) have been found to be predictors of emotional exhaustion [35]. Nevertheless, although the role of anxiety sensitivity on other organizational results, such as engagement, has been analyzed elsewhere and found to be linked specifically to less job commitment mediated by psychological acceptance [36], as far as we know, there are very few studies on the relationship between anxiety sensitivity and school burnout.

### 1.3. Burnout and Anxiety Sensitivity: Importance of Emotional Intelligence

Even though continuous exposure to school-related stressors often leads to burnout, the presence of some individual traits and characteristics has been shown to act in this relationship, leading to different degrees of vulnerability to this syndrome in adolescence [37,38]. Individual variables such as emotional skills have a fundamental role in students’ school life. School-related emotion regulation has been shown to predict exhaustion levels [39] and is associated with student academic success, self-efficacy, and well-being [40,41,42]. Emotional intelligence refers to the perception, comprehension, and effective management of one’s own and others’ emotions [43].

Of the factors that comprise this construct, mood and stress management factors have been shown to be especially relevant in preventing the development of burnout in the working population [44,45]. Similarly, a study by Erbíl et al. [46] found a strong negative association between stress management and emotional intelligence mood state with burnout levels in architecture students. In school, high levels of emotional intelligence have been associated with less academic stress [47]. Thus, students with more emotional intelligence can fight academic stress effectively, increasing their satisfaction with life [48] and commitment and performance in the classroom [49,50]. In fact, a study of Spanish high school students found a negative relationship between emotion regulation and the rest of the emotional intelligence factors with academic burnout [51]. On the contrary, poor emotion regulation increases burnout in adolescents [39].

Therefore, working on emotional competency in schools may be an effective measure for decreasing the exhaustion of high school students [52]. Along this line, as demonstrated by Cheung and Li [53], the mental strength of youths is related to academic exhaustion, such that the ability to remain under control and focused under stress in stressful situations is associated with less burnout. However, the study by Caballero-Domínguez and Suárez-Colorado [54] showed that “burnt” students (that is, those who have high scores in Exhaustion and Cynicism and low scores in Efficacy) show high levels of anxiety and do not employ adaptive strategies for alleviating their distress, such as expressing their emotions.

According to the discussion above, emotional intelligence can exert a protective effect when the symptomatic and emotional manifestations of anxiety in everyday stressful events acquire the connotation of catastrophic, that is, when youths have high levels of anxiety sensitivity [55]. This hypothesis is supported by studies such as the one by Sahranavard et al. [56], who found that improving stress management (one of the emotional intelligence factors) of students was effective in reducing anxiety sensitivity, which increased their ability to cope with academic challenges and their expectations for educational success. In this regard, a high school program in which adolescents who were taught skills for reducing anxiety by managing their emotions, among other factors, improved their functioning and daily well-being, in addition to preventing the appearance of mental health problems negatively affecting school performance, such as burnout [57]. Thus, caring for the feelings and emotions of students with school-related problems has demonstrated improvement in emotional alterations, preparing them to cope with various academic situations [56]. This support in the academic context is especially necessary for students prone to anxiety [58] since providing individuals with a greater capacity for managing their emotional states with emotional intelligence enables anxiety to be reduced, and this then has repercussions on their well-being [59] and their academic performance [60].

### 1.4. The Present Study

Several studies in fields such as health sciences have emphasized the recurrence of stress as a risk factor for developing academic burnout [61,62,63]. It is also widespread in university and secondary education students, where the impact of continuous academic stress on the results and well-being of students has not yet been explored in detail [64]. Research in this area is therefore necessary, more so from a positive approach to stress management as a protective emotional factor intervening in the prevention of academic burnout. Academic burnout is the reaction of an individual to stressors in the student environment. However, not all students in the same academic environment develop burnout. This could be related to personality characteristics, such as proneness to anxiety [65], so we intended to find out whether anxiety sensitivity is a factor directly related to burnout.

This study was further based on the concept of emotional intelligence as a series of competencies and skills in understanding, expressing, and coping with emotions [66]. These emotional intelligence skills and competencies are thought to be necessary for success in work and school [67]. Emotional intelligence enables immediate situations to be coped with realistically and flexibly, solving problematic situations successfully, and enabling personal and environmental change for success in life [66]. Thus, emotional intelligence could be a factor involved in the relationship between anxiety sensitivity and academic burnout. Especially relevant in this association are stress management factors and emotional intelligence mood state. Intervention in stress management has been effective in reducing academic stress and improving the well-being of university students [68]. Along with stress management, mood is also present in the scientific literature as another of the emotional factors related to students’ academic well-being [46,69]. Moreover, in some proposed intervention programs, both variables are starting to be taken together [70].

This study widened research on individual differences in anxiety sensitivity and problems at school, specifically academic burnout, by examining the mediating role of emotional intelligence. Anxiety sensitivity is associated with perceived threat, so we hypothesized that this could generate greater stress and exhaustion in students from academic events, favoring the appearance of burnout. We also thought that emotional intelligence could have an important role in this relationship, and in particular, stress management and mood factors would affect it directly. Higher levels of these factors would help control adverse emotional symptoms of anxiety sensitivity, and finally, they would be linked to less academic burnout.

In view of all of the above, we formulated the following hypotheses: Anxiety sensitivity of high school students affects academic burnout (H1); Emotional intelligence protects them in stressful situations (H2). Insofar as we know, the relationship between these variables has not yet been studied in an adolescent population. Therefore, the first objective of this study was to identify the relationship between anxiety sensitivity, academic burnout, and emotional intelligence in high school students, and in addition, to determine the existence of differences in burnout levels depending on their anxiety sensitivity and any possible influence of emotional intelligence.

## 2. Materials and Methods

### 2.1. Participants

A total of 1287 students, aged 14 to 18, with a mean age of 15.11 (*SD* = 0.91), participated in this study. They were selected by simple random sampling from 11 public high schools chosen using cluster sampling in the province of Almería (Spain), all of them in urban areas. Of these, 47.1% (*n* = 606) were boys and 52.9% (*n* = 681) were girls, with a mean age of 15.12 (*SD* = 0.94) and 15.10 (*SD* = 0.88), respectively; 55% (*n* = 707) were in 3rd year and 45% (*n* = 577) in 4th year.

### 2.2. Procedure

This cross-sectional study was approved by the Bioethics Committee of University of Almeria (Ref: UALBIO2018/015). The questionnaires, which took 20–25 min to complete, were administered individually and anonymously in ordinary classrooms. The students’ teachers were present in the classroom. To contact the participants, a meeting was first held with the high school principal, where the objectives of the study were explained, and appropriate permission was requested. In all cases, the confidentiality of data and compliance with ethical standards were guaranteed, and the informed consent of the students’ parents was received. The Strengthening the Reporting of Observational Studies in Epidemiology (STROBE) [71] guidelines were followed throughout the study.

### 2.3. Measures

An ad hoc questionnaire was prepared for sociodemographic information, such as the age and sex of the participants.

The Maslach Burnout Inventory-General Survey [72] student version (MBI-SS) [73] was used to evaluate burnout. This instrument consists of 15 items rated on a Likert-type scale (where 1 is “never”) and 6 is “always/every day”), which evaluate the feelings and attitudes of students toward their school activity through three factors: emotional exhaustion, cynicism, and inefficacy. The first factor evaluates the feeling of physical, mental, and emotional exhaustion, the impossibility of being able to do anything else to cope with school demands. The cynicism factor reflects criticism of the usefulness of their studies. Efficacy refers to feeling competent to study. Reliability in this study was as follows: Cynicism: α = 0.818, ω = 0.827, GLB = 0.853; Efficacy: α = 0.793, ω = 0.796, GLB = 0.797.

The adapted Spanish version for high school students of the Anxiety Sensitivity Index-3 (ASI-3) [74,75] was used to evaluate anxiety sensitivity. This questionnaire is made up of 18 items with a Likert-type response format (where 0 equals “very little” and 4 “very much”). It consists of three subscales: physical, cognitive, and social anxiety sensitivity. The scale’s reliability in this study was: Cognitive: α = 0.853, ω = 0.856, GLB = 0.885; Social: α = 0.781, ω = 0.788, GLB = 0.794; Physical: α = 0.860, ω = 0.862, GLB = 0.852. 

Emotional intelligence was evaluated using the *Brief Emotional Intelligence Inventory* (EQ-i-M20) [76]. This instrument consists of 20 items for calculating 5 factors (intrapersonal, interpersonal, stress management, adaptability, and general mood). These factors refer to the capacity for understanding one’s own feelings and the emotions of others, the ability to maintain self-control in distressful situations, flexibility in resolving problems, and feelings of happiness and optimism, respectively. Items are answered on a four-point Likert-type scale (from “never happens to me” to “always happens to me”). The reliability in this study was: Intrapersonal: α = 0.814; ω = 0.818, GLB = 0.820; Interpersonal: α = 0.584, ω = 0.619, GLB = 0.680; Stress management: α = 0.770, ω = 0.773, GLB = 0.801; Adaptability: α = 0.713, ω = 0.720, GLB = 0.734; Mood: α = 0.870, ω = 0.871, GLB = 0.878.

### 2.4. Statistical Analysis

First, to identify the relationship between the variables, a bivariate correlation analysis was computed with Pearson’s correlation coefficient. Participants were statistically classified by their mean scores on each of the anxiety sensitivity factors by two-stage cluster analysis for distribution into two clusters or groups. Where the differences were not statistically significant or had a tendential *p*-value, the Bayesian alternative was computed, which enabled the evidence of the null hypothesis to be compared (H_0_: there are no differences between groups) to the alternative hypothesis (H_1_: there are differences between groups). The Bayesian *t* was estimated using JASP statistical software ver. 0.11.1 [77]. The Cauchy prior width was set as predetermined by the software at 0.707.

The emotional intelligence components which correlated to the three anxiety sensitivity factors were extracted from the correlation matrix results, and in turn, with the academic burnout dimensions. Then, the mediation models were computed with anxiety sensitivity as the predictor variable, the components of emotional intelligence as mediators, and the dimensions of academic burnout as the dependent variables. The multiple mediation models were computed using the PROCESS macro for SPSS [78], with 5000 bootstrapping samples. Following recent recommendations for estimating the statistical power of mediation models [79], effect size was determined by Monte Carlo Power Analysis for Indirect Effects, with correlations as the input method.

To examine the reliability of the instruments used for data collection, McDonald’s Omega coefficient [80] was estimated, following the proposal and recommendations of Ventura-León and Caycho [81].

## 3. Results

### 3.1. Correlations and Descriptive Analyses

As observed in the correlation matrix (Table 1), exhaustion was negatively related to the Intrapersonal, Stress Management, and Mood components. The Interpersonal factor was positively correlated with it, but weakly, while the Cynicism factor correlated negatively with the Intrapersonal, Stress Management, Adaptability, and Mood factors.

The relationships with all three anxiety sensitivity factors were positive with both Exhaustion and Cynicism.

Finally, Efficacy was positively correlated to all the components of emotional intelligence, while it was weakly negatively related to two of the factors of anxiety sensitivity (cognitive and social).

A two-stage cluster analysis was performed for participant distribution into two groups or clusters by anxiety sensitivity. The resulting composition of the two groups was the following:

Cluster 1, made up of 72.8% (*n* = 937) of the sample, contained participants with mean scores on anxiety sensitivity below the total sample mean. Cluster 2, with the remaining 27.2% (*n* = 350) of the sample, was made up of participants with mean scores in anxiety sensitivity above the total sample mean.

Table 2 shows the descriptive statistics of the total sample and both clusters and also the results of the *t*-test for independent samples. Statistically significant differences between the groups can be seen in the Exhaustion and Cynicism dimensions. In both cases, the group with the higher mean scores in anxiety sensitivity had the highest scores in these two dimensions of burnout.

**Figure 1:**
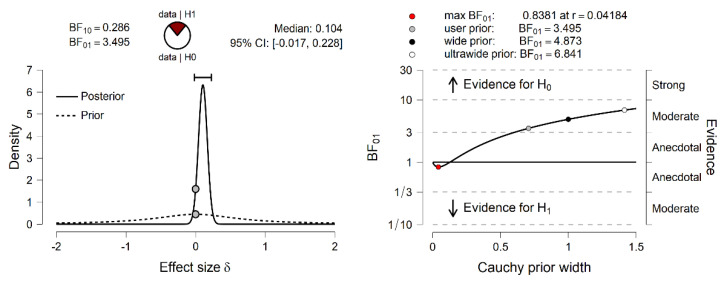
Efficacy. Bayesian Independent Samples *t*-Test [Inferential plots: (**Left panel**) Prior and Posterior; (**Right panel**) Bayes Factor Robustness Check].

No significant differences in Efficacy were observed between the low/high anxiety sensitivity groups. To test the evidence in favor of H_0_ against H_1_, the BF_01_ was computed (Bayes factor in favor of H_0_ over H_1_). The Cauchy prior width was set at its JASP predetermined value of r = 0.707. As observed on the left of Figure 1, it is obvious that most of the posterior distribution is positive. The posterior median was 0.104, which varied from −0.017 to 0.228, and 95% CI. The BF_01_ was 3.49, which indicates that the data observed were 3.49 times more likely under H_0_ than under H_1_.

### 3.2. Analysis of the Mediating Effect of Emotional Intelligence on the Predictive Value of Anxiety Sensitivity for Academic Burnout

**Figure 2:**
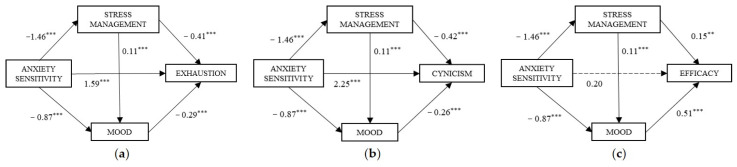
Statistical diagrams of the direct effects on the mediation models proposed. [Note. (**a**) Anxiety sensitivity and Exhaustion; (**b**) Anxiety sensitivity and Cynicism; (**c**) Anxiety sensitivity and Efficacy. ** *p* < 0.01, *** *p* < 0.001].

A multiple mediation model was proposed for each academic burnout factor, with two mediator variables. Both stress management and mood were entered in the models as Mediator 1 and Mediator 2, respectively. In all cases, the anxiety sensitivity level (low/high), coinciding with the groups extracted from the cluster analysis above, was included as the predictor variable (Figure 2).

In the first place, a significant effect of anxiety sensitivity on stress management, *β* = −1.46, 95% IC (−1.81, −1.12), and mood, *β* = −0.87, 95% IC (−1.27, −0.47), is observed. The effect on the relationship between mediators was positive *β*= 0.11, 95% IC (0.05, 0.17). 

In addition, the effects of the mediators on the dependent variable were: (a) a significant effect of stress management, *β* = −0.41, 95% IC (−0.53, −0.30), and mood *β* = −0.29, 95% IC (−0.39, −0.19) on exhaustion; (b) both stress management, *β* = −0.42, 95% IC (−0.54, −0.29), and mood, *β* = −0.26, 95% IC (−0.37, −0.15), had significant effects on cynicism; and (c) stress management, *β* = 0.15, 95% IC (0.05, 0.24), and mood, *β* = 0.51, 95% IC (0.43, 0.59), showed significant direct effects on efficacy.

Moreover, as shown in Table 3, based on the analysis of indirect effects with bootstrapping, the following results were found for each model: 

Model (a): The total indirect effect was *β* = 0.91, 95% IC (0.65, 1.21), where the three paths were significant, and path IE_1_ (Anxiety sensitivity → Stress management → Exhaustion) had the strongest effect. Estimation of the statistical power of the indirect effects using the Monte Carlo simulation showed 1.00 for IE_1_, 0.92 for IE_2_ (Anxiety sensitivity → Stress management → Mood → Exhaustion), and 0.99 for IE_3_ (Anxiety sensitivity → Mood → Exhaustion).

Model (b): The total indirect effect was *β* = 0.89, 95% IC (0.64, 1.19), where the three paths were significant, and path IE_1_ (Anxiety sensitivity → Stress management → Cynicism) was the most representative. Monte Carlo simulation showed a statistical power of 1.00 for IE_1_, 0.93 for IE_2_, and 0.99 for IE_3_.

Model (c): For this model, which takes efficacy as the dependent variable, a nonsignificant direct effect and a tendential total effect should be emphasized (# *p*-value over 0.05 but below 0.10). However, the total indirect effect was *β* = −0.75, 95% IC (−1.04, −0.48). A priori, all three paths seemed to be significant; however, after comparing mediators, statistically significant differences were found in Comparison 3 [C3: 0.36, SE = 0.12, 95% IC (0.13, 0.62)], which compared the indirect effects of path IE_2_ (Anxiety sensitivity → Stress management → Mood → Efficacy) minus IE_3_ (Anxiety sensitivity → Mood → Efficacy). The positive sign in the coefficient of C3 shows that the indirect effect of path IE_2_ (Anxiety sensitivity → Mood → Efficacy) is greater than path IE_3_. Therefore, it may be said that the indirect effect of sensitivity on anxiety is greater with the two mediators operating in series. The statistical power found with Monte Carlo simulation was 0.90 for IE_1_, 0.92 for IE_2_, and 0.99 for IE_3_.

## 4. Discussion

The educational context of high school often generates overload and exposure of adolescents to highly stressful situations [5], which can lead to exhaustion and academic burnout [6,7]. Due to the need to improve the well-being and adaptation of youths in this stage of their education [2], the objective of this study was to establish the relationship between individual factors, such as anxiety sensitivity and emotional intelligence, in the appearance of this syndrome.

The results showed positive correlations between exhaustion and cynicism and physical, cognitive, and social anxiety sensitivity, while for efficacy, they were negative. These results, therefore, suggest a relationship between more vulnerability to anxiety and academic burnout. This is especially important because, insofar as we know, the association between these variables has been analyzed very little, although previous studies have already pointed in this direction by showing the presence of stronger symptoms of academic burnout in students with anxiety symptoms [32,33,57]. At the same time, the efficacy factor of burnout was found to correlate positively with all the emotional intelligence scales. However, in emotional exhaustion, the relationships were negative for the intrapersonal, stress management, and mood components of emotional intelligence, to which adaptability was added in the case of cynicism. These data are similar to those found in other studies, which have shown negative relationships between emotion regulation and academic burnout levels [46].

Then, the sample was clustered by anxiety sensitivity levels to find out whether more vulnerability to adverse events made a difference in the students’ burnout levels. According to the results, the youths in the group with high anxiety sensitivity showed higher levels of emotional exhaustion and cynicism than students with low sensitivity, while no differences were found in efficacy. These results show that adolescents who interpreted the symptoms of anxiety as potentially dangerous tended to show an attitude of detachment from their studies and emotional exhaustion. Nevertheless, they did not feel their academic performance was any less effective than other students who were less sensitive to anxiety. This finding can be interpreted in the light of experiential avoidance studies [27,29], which have postulated that individuals with high anxiety sensitivity avoid situations that make them nervous, such as stressful academic events [24]. Thus, facing situations at school as if they were valueless and personally weakening would make the stress generated by stressful academic events intimidating, without this implying that their feeling of academic capability was reduced. We may therefore say that anxiety sensitivity is a personality factor associated with academic burnout, and that stress management and mood, as components of emotional intelligence, intervene in this relationship by meditating between the two variables. Students who are not as able to cope with overwhelming emotions and who have a negative mood would have a greater feeling of threat from stimuli identified as adverse, and finally, they would have higher levels of burnout. Nevertheless, the model proposed and the direction of the relationship between the variables should be tested in longitudinal studies.

Finally, with the results found up to then, an analysis was made of the possible mediating effect of emotional intelligence on the predictive power of anxiety sensitivity for academic burnout. Specifically, stress management and mood, as the emotional intelligence factors correlated with all the academic burnout subscales and anxiety sensitivity, were entered as possible mediators. The mediation analysis showed that high anxiety sensitivity directly affected student exhaustion, cynicism, and efficacy levels. Although these effects were mainly mediated by stress management, they were also mediated through the effect of stress management on mood. Other studies have shown how stress management is related negatively to academic burnout [51] by enabling students to fight stressful situations [48]. Moreover, studies carried out in working populations have identified the importance of the mood and stress management factors of emotional intelligence in preventing burnout [44,45]. This study assumed that these emotional intelligence dimensions also exert a protective effect on the development of academic burnout when young people who face stressful experiences are especially vulnerable to the symptoms of anxiety.

In view of the above, working with emotional competencies in schools would be an effective measure for protecting against the development of exhaustion and diminishing its levels in high school [52], as it is an adaptive strategy [54] that enables emotions derived from high anxiety sensitivity to be kept under control [55], especially under pressure [53]. As mentioned above, anxiety sensitivity is an individual characteristic [65] that causes stressful events in the school context to have a stronger effect on developing burnout. The results of this study suggest the mediating role of stress management and mood factors in the relationship between anxiety sensitivity and burnout. Thus, according to Bar-On’s concept of emotional intelligence as a skill [66], this competence should be improved and trained, making it easier for the most vulnerable individuals to adapt, thereby reducing academic burnout levels. In this regard, other studies on emotional intelligence training for university students have been shown to be effective in improving this skill and mitigating the effects of exhaustion [82]. We suggest the need for future studies on whether intervention in emotional skills for secondary students reduces burnout in those most sensitive to anxiety.

### Limitations and Future Directions

Finally, some limitations should be mentioned. In the first place, the cross-sectional design did not allow us to establish any causal relationships between variables. This is particularly important in interpreting the results of the mediation analysis, which was based on relationships between variables as a potential alternative to the causal inference methods, and must therefore be taken with caution [83]. Our study evaluated all the preliminary requirements necessary for mediation analysis. However, in spite of this, our focus was based on cross-sectional data that can lead to distorted conclusions if compared with longitudinal research [84]. Further prospective studies on the subject should be encouraged.

The scant bibliography found for analyzing the relationship between the variables in this study in high school students impeded the construction of a sufficient theoretical foundation to back up our findings. Therefore, a more profound study of the relationship between anxiety sensitivity, emotional intelligence, and academic burnout in high school students would be favorable in this area of science. Furthermore, environmental factors that may have been affecting the participants were not taken into consideration. Thus, the variables analyzed were individual, and other elements, such as exposure to stressful situations, were not considered. Given the importance of sustained stress in developing burnout and that anxiety sensitivity becomes especially visible when individuals are exposed to events that cause them to worry, future studies should include this variable.

We should also point out that this study’s objective was only a first approach to the analysis of the relationships between the variables. Future research should go deeper into more complex associations and propose a model that provides more specific data on their validity by selecting instruments that can find latent variables, for example.

## 5. Conclusions

High school can mean large academic changes for adolescents that can impede their classroom engagement and performance. The high presence of academic burnout in high schools is a severe social concern because of its effect on various risk variables in young people. Knowing how individual variables such as anxiety sensitivity and emotional intelligence influence the development of this syndrome would increase progress in approaching exhaustion. Specifically, this study demonstrated that high anxiety sensitivity affects burnout levels in young people and that stress management and mood, both emotional intelligence factors, mediate in this relationship. In conclusion, despite the fact that education laws defend the promotion of emotional education in secondary education, more specific programs for developing emotional intelligence are still necessary.

Therefore, promoting the development of emotional skills that enable managing emotions when they become overwhelming, especially among highly vulnerable young people, could lessen the lack of interest and exhaustion in the classroom, improving the performance and well-being of students in adolescence.

## Figures and Tables

**Table 1:** Bivariate correlation matrix.

		EXH		CYN		EFF		INTRA		INTER		STR_M		ADAPT		MOOD		ANXc		ANXs		ANXp	
EXH	Pearson’s r	—																					
	Upper 95% CI	—																					
	Lower 95% CI	—																					
CYN	Pearson’s r	0.592	***	—																			
	Upper 95% CI	0.626		—																			
	Lower 95% CI	0.555		—																			
EFF	Pearson’s r	−0.126	***	−0.234	***	—																	
	Upper 95% CI	−0.071		−0.182		—																	
	Lower 95% CI	−0.179		−0.285		—																	
INTRA	Pearson’s r	−0.090	**	−0.075	**	0.161	***	—															
	Upper 95% CI	−0.036		−0.020		0.214		—															
	Lower 95% CI	−0.144		−0.129		0.107		—															
INTER	Pearson’s r	0.065	*	0.009		0.190	***	0.207	***	—													
	Upper 95% CI	0.119		0.063		0.242		0.259		—													
	Lower 95% CI	0.010		−0.046		0.137		0.154		—													
STR_M	Pearson’s r	−0.242	***	−0.238	***	0.121	***	−0.034		−0.013		—											
	Upper 95% CI	−0.190		−0.186		0.175		0.021		0.042		—											
	Lower 95% CI	−0.293		−0.289		0.067		−0.088		−0.067		—											
ADAPT	Pearson’s r	0.007		−0.066	*	0.323	***	0.253	***	0.408	***	−0.046		—									
	Upper 95% CI	0.062		−0.011		0.371		0.304		0.452		0.009		—									
	Lower 95% CI	−0.048		−0.120		0.273		0.202		0.361		−0.101		—									
MOOD	Pearson’s r	−0.195	***	−0.177	***	0.330	***	0.376	***	0.140	***	0.128	***	0.343	***	—							
	Upper 95% CI	−0.142		−0.124		0.378		0.422		0.193		0.181		0.391		—							
	Lower 95% CI	−0.247		−0.230		0.280		0.328		0.086		0.074		0.294		—							
ANXc	Pearson’s r	0.220	***	0.263	***	−0.063	*	0.004		0.055	*	−0.256	***	−0.016		−0.136	***	—					
	Upper 95% CI	0.271		0.313		−0.009		0.059		0.110		−0.205		0.039		−0.081		—					
	Lower 95% CI	0.167		0.211		−0.117		−0.050		0.001		−0.307		−0.071		−0.189		—					
ANXs	Pearson’s r	0.242	***	0.222	**	−0.061	*	−0.084	**	0.076	**	−0.236	***	−0.014		−0.182	***	0.606	***	—			
	Upper 95% CI	0.293		0.273		−0.006		−0.030		0.130		−0.184		0.040		−0.128		0.640		—			
	Lower 95% CI	0.190		0.169		−0.115		−0.138		0.021		−0.287		−0.069		−0.234		0.570		—			
ANXp	Pearson’s r	0.207	***	0.195	***	−0.003		0.043		0.103	***	−0.238	***	−0.009		−0.068	*	0.671	***	0.515	***	—	
	Upper 95% CI	0.259		0.247		0.052		0.097		0.157		−0.186		0.046		−0.013		0.700		0.554		—	
	Lower 95% CI	0.155		0.142		−0.058		−0.012		0.049		−0.289		−0.063		−0.122		0.640		0.473		—	

Note. EXH = Exhaustion, CYN = Cynicism, EFF = Efficacy, INTRA = Intrapersonal, INTER = Interpersonal, STR_M = Stress management, ADAP = Adaptability, MOOD = Mood, ANXc = Anxiety cognitive factor, ANXs = Anxiety social factor, ANXp = Anxiety physical factor. * *p* < 0.05, ** *p* < 0.01, *** *p* < 0.001.

**Table 2:** Differences in burnout by level of anxiety sensitivity.

Burnout	Group	*N*	*M*	*SD*	*t*	*p*	*d*
EXH	ANX Low	937	13.88	6.13	−6.64 ***	0.000	−0.41
	ANX High	350	16.40	5.79			
	Total sample	1287	14.57	6.14			
CYN	ANX Low	937	9.45	6.39	−7.91 ***	0.000	−0.49
	ANX High	350	12.59	6.23			
	Total sample	1287	10.30	6.50			
EFF	ANX Low	937	14.64	5.13	1.69	0.091	-
	ANX High	350	14.09	5.29			
	Total sample	1287	14.49	5.18			
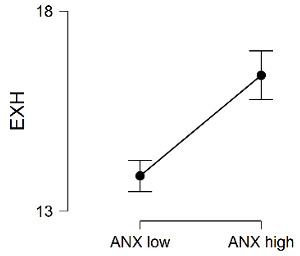			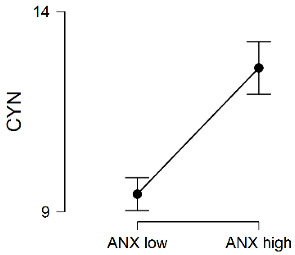			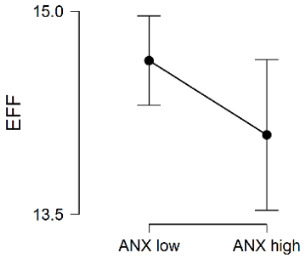	

Note. EXH = Exhaustion, CYN = Cynicism, EFF= Efficacy, ANX = Anxiety sensitivity. *** *p* < 0.001.

**Table 3:** Direct, total, and indirect effects.

**(a) Anxiety Sensitivity and Exhaustion**	***β***	**SE**	**t**	**95% CI**
Direct effect: Anxiety sensitivity → Exhaustion	1.59 ***	0.37	4.22	(0.85, 2.33)
Total effect: Anxiety sensitivity → Exhaustion	2.51 ***	0.37	6.64	(1.77, 3.25)
IE 1: Anxiety sensitivity → Stress management → Exhaustion	0.61	0.11		(0.41, 0.84)
IE 2: Anxiety sensitivity → Stress management → Mood → Exhaustion	0.04	0.01		(0.01, 0.09)
IE 3: Anxiety sensitivity → Mood → Exhaustion	0.25	0.07		(0.12, 0.45)
**(b) Anxiety sensitivity and Cynicism**	***β***	**SE**	**t**	**95% CI**
Direct effect: Anxiety sensitivity → Cynicism	2.25 ***	0.39	5.64	(1.47, 3.04)
Total effect: Anxiety sensitivity → Cynicism	3.14 ***	0.39	7.91	(2.36, 3.92)
IE 1: Anxiety sensitivity → Stress management → Cynicism	0.61	0.11		(0.41, 0.88)
IE 2: Anxiety sensitivity → Stress management → Mood → Cynicism	0.04	0.01		(0.01, 0.08)
IE 3: Anxiety sensitivity → Mood → Cynicism	0.23	0.07		(0.11, 0.41)
**(c) Anxiety sensitivity and Efficacy**	***β***	**SE**	**t**	**95% CI**
Direct effect: Anxiety sensitivity → Efficacy	0.20	0.31	0.66	(−0.41, 0.83)
Total effect: Anxiety sensitivity → Efficacy	−0.54 ^#^	0.32	−1.68	(−1.18, 0.08)
IE 1: Anxiety sensitivity → Stress management → Efficacy	−0.22	0.08		(−0.39, −0.07)
IE 2: Anxiety sensitivity → Stress management → Mood → Efficacy	−0.08	0.03		(−0.15, −0.03)
IE 3: Anxiety sensitivity → Mood → Efficacy	−0.45	0.11		(−0.69, −0.23)

Note. IE = Indirect effect, SE = Standard Error, CI = Confidence interval. Sample size Bootstrap for Indirect Effects = 5000; *β* = non-standardized regression coefficient; *** *p* < 0.001, ^#^ 0.05 < *p* < 0.10.

## Data Availability

The data that support the findings of this study are available from the corresponding author upon reasonable request.
